# Supplementary material for: Angelica sinensis polysaccharide as potential protectants against recurrent spontaneous abortion: focus on autophagy regulation
Source: Front Med (Lausanne). 2025 Jan 15;12:1522503. doi: 10.3389/fmed.2025.1522503 (PMC11774876; doi:10.3389/fmed.2025.1522503)
Supplement: Supplementary material S1 — The main instruments used during the LC-MS process, along with their models/specifications and manufacturers. [file Supplementary_file_1.zip › Metabolomics sequencing data FC1.2/8.代谢通路分析/Exposure_Ctrl/all/KEGG/mmu04976.html]

KEGG PATHWAY: Bile secretion - Mus musculus (house mouse)


# Bile secretion - Mus musculus (house mouse)


[
Pathway menu
| Organism menu
| Pathway entry
| Show description
| Download
| Help
]

Bile is a vital secretion, essential for digestion and absorption of fats and fat-soluble vitamins in the small intestine. Moreover, bile is an important route of elimination for excess cholesterol and many waste product, bilirubin, drugs and toxic compounds. Bile secretion depends on the function of membrane transport systems in hepatocytes and cholangiocytes and on the structural and functional integrity of the biliary tree. The hepatocytes generate the so-called primary bile in their canaliculi. Cholangiocytes modify the canalicular bile by secretory and reabsorptive processes as bile passes through the bile ducts. The main solutes in bile are bile acids, which stimulate bile secretion osmotically, as well as facilitate the intestinal absorption of dietary lipids by their detergent properties. Bile acids are also important signalling molecules. Through the activation of nuclear receptors, they regulate their own synthesis and transport rates.


##### Option

Scale:


100%

Image resolution:


 High

##### Search

##### ID search

##### Color


KGML

Image (png) file 1x
